# Extraction Optimization of *Quercus cerris* L. Wood Chips: A Comparative Study between Full Factorial Design (FFD) and Artificial Neural Network (ANN)

**DOI:** 10.3390/antiox13091115

**Published:** 2024-09-14

**Authors:** Maria Ponticelli, Vittorio Carlucci, Marisabel Mecca, Luigi Todaro, Luigi Milella, Daniela Russo

**Affiliations:** 1 Department of Science, University of Basilicata, Via dell’Ateneo Lucano 10, 85100 Potenza, Italy; maria.ponticelli@unibas.it (M.P.); vittorio.carlucci@unibas.it (V.C.); daniela.russo@unibas.it (D.R.); 2 Department of Biochemical Pharmacology & Drug Design, Institute of Molecular Biology “Roumen Tsanev”, Bulgarian Academy of Sciences (BAS), Acad. G. Bonchev Str., bl. 21, 1113 Sofia, Bulgaria; 3 Laboratory of Preclinical and Translational Research, Centro di Riferimento Oncologico della Basilicata (IRCCS-CROB), 85028 Rionero in Vulture, Italy; marisabelmecca@libero.it; 4 School of Agriculture, Forest, Food and Environmental Sciences, University of Basilicata, Via dell’Ateneo Lucano 10, 85100 Potenza, Italy; luigi.todaro@unibas.it; 5 BioActiPlant s.r.l., Via dell’Ateneo Lucano 10, 85100 Potenza, Italy

**Keywords:** *Quercus cerris* L., wood chips, antioxidant activity, full factorial design, artificial neural networking, specialized metabolites

## Abstract

From a circular bio-economy perspective, biomass valorization requires the implementation of increasingly efficient extraction techniques to ensure the environmental and economic sustainability of biorefining processes. This research focuses on optimizing the specialized metabolite extraction of Turkey oak chips from *Quercus cerris* L. by applying a 3 levels Full Factorial Design (FFD). The goal is to obtain an extract with the highest antioxidant activity [evaluated by 1,1-diphenyl-2-picryl hydrazyl (DPPH) scavenging activity and ferric reducing antioxidant power (FRAP) assays] and specialized metabolites content [measured as total phenolic content (TPC), total flavonoid content (TFC), condensed tannin content (CTC), and hydrolysable tannins content (THC)]. With this objective, three different variables were investigated and compared: temperature (20 °C, 50 °C, 80 °C), solvents EtOH/H_2_O (0%, 20%, 40%), and time (3 h, 6 h, 24 h), resulting in 27 different extracts. Following the FFD analysis, the optimal extractive conditions were determined to be 80 °C, 40% EtOH/H_2_O, and 19.8 h. Finally, the prediction ability of FFD was compared with that of artificial neural network (ANN) for DPPH scavenging activity, FRAP, and TPC data based on the coefficient of determination (R^2^), mean absolute error (MAE), and root mean square error (RMSE). The results indicated that ANN predictions were more precise than FFD ones; however, both methods were useful in optimizing the extraction process as they returned comparable optimized extraction parameters.

## 1. Introduction

On a global scale, significant quantities of wood residues stem from forest management practices; however, their utilization is limited due to challenges such as geographical distances, inaccessibility, and the economic expenses involved in their collection and processing. Another important sector of wood by-product sources is the manufacturing field, which leads to the production of wood sawdust, chips, and shavings [1,2]. Therefore, applying the concept of circular economy may represent a valuable framework for advancing towards a future green economy by reducing waste, especially within the context of the wood industry. Currently, wood-derived biomass represents the foremost renewable energy source globally and also finds application in producing wood–plastic composites through wood chips and sawdust [1]. Another use of particular interest is the recovery of bio-functional molecules from wood waste for reuse and application in various fields like pharmaceuticals, cosmetics, and food [3]. Considering this point, enhancing existing phytochemical extraction protocols or developing new ones emerge as matters of practical significance in the context of wood biomass recirculation. Hence, the present investigation aims to develop a new extractive protocol for *Quercus cerris* L. wood chips using environmentally friendly protocols that align with the green economy concept.

Turkey oak (*Quercus cerris* L.) is an important forest species that is widespread in the South-Eastern Mediterranean countries [4]. Specifically, it ranges from Southern Europe to Asia Minor, with greater concentration in the Balkan peninsula and Italy [5]. Turkey oak represents one of the five most important deciduous oaks for the Italian flora, together with oak and farnetto (*Quercus frainetto* Ten.) [6]. In this respect, Turkey oak covers a substantial portion of the European forest area and holds considerable economic importance, particularly for communities in marginal mountain zones. Considering its qualitative properties and technological performances (e.g., low dimensional stability, prone to cracking, different technological properties between heartwood and sapwood, etc.), the Turkey oak wood is poorly appreciated for industrial application, but widely used for energetic purposes [7]. Simultaneously, the extraction of secondary and non-structural metabolites of plant cells that can be used in the medical, nutraceutical, and industrial fields are being investigated as products of an integrated biorefinery with high added value. Several studies reported the secondary metabolite profile and bioactivity of extracts from *Q. cerris* trees [8]. For instance, Cetera et al. have recently shown that subjecting Turkey oak wood to thermal treatment results in enhanced extraction yields when employing maceration, ultrasound-assisted extraction, and accelerated solvent extraction (ASE) techniques, leading to increased levels of polyphenols and flavonoids [9]. The composition of extractives varies among species, since the total amount of extractives is influenced by several factors such as genetic origin, collection of raw materials, and the environmental conditions of the growth locations. For this reason, despite possible phenological and taxonomic similarities, differences have been evaluated even between closely related species. In particular, secondary metabolites are found in greatest quantities in the bark, leaves, and roots, with significant differences between the different parts of the same plant. The bark contains a large number of phenolic molecules, such as condensed tannins, monomeric flavonoids, lignans, and stilbenes, known to be involved in several processes like biosynthesis, nutrient reserve, protection from fungal and insect attacks, and maintenance of physiological balance. Furthermore, they also contribute to wood properties such as color, odor, and flavor [10,11]. 

Despite its widespread distribution, Turkey oak is often utilized primarily as firewood. However, expanding its industrial applications and exploring the potential use of its by-products as a source of antioxidants could significantly increase the economic value of this crop and further enhance its utility. It is for these reasons that the valorization of secondary wood products is still an open challenge of great interest. Given that the extraction process has been shown to influence the yield, phytochemical composition, and biological properties of extractives, this study aimed to investigate the combined effects that variations in temperature, solvents, and extraction time can have on the extraction processes of *Quercus cerris* L. wood chips. Therefore, a three-level full factorial design (FFD) and artificial neural networking (ANN) were applied to evaluate the optimal operating conditions to obtain extract containing the highest content of specialized metabolites with antioxidant activity. Several studies have been conducted on the antioxidant activity and content of polyphenols, flavonoids, and tannins of Turkey oak wood using different extraction techniques such as microwave-assisted extraction (MAE) or maceration coupled with different solvents, including pure water; 100% acetone; 75% EtOH; 75% MeOH; and 75, 50, 25% acetone [12,13,14]. However, no studies have been conducted on the extraction optimization of *Q. cerris* wood chips using easy and industrially scalable protocols, which include maceration and digestion as extractive methods and safe solvents such as EtOH/H_2_O. According to Luís et al. the mixture of water and ethanol is the most appropriate for extracting chemical compounds from plant materials with biological activity, and this finding is strongly supported by the evidence that they may represent an eco-friendly solution without negative impacts on human health and the environment [15]. Among the various extraction methods, a particularly advantageous, affordable, and easily scalable process has been chosen for industrial applications: maceration and digestion, since these processes align with the principle of green chemistry and with the goal of a sustainable economy [16]. Therefore, by opting for these methods and environmentally friendly solvents like water and ethanol, the extraction of specialized metabolites from Turkey oak wood chip will be optimized in full compliance with green economy directives; also, the environmental impact will be minimized, resulting in the application of low-environmental-impact experimental methodologies. This decision underscores a strategic commitment to sustainable practices within the industry, showcasing a dedication to waste reduction and promoting an eco-friendly production process beyond the simple recovery of bioactive compounds from wood chip waste.

## 2. Materials and Methods

### 2.1. Chemicals

The reagents 2,4,6-tripyridyl-s-triazine (TPTZ), iron (III) chloride (FeCl_3_∙6H_2_O), Folin–Ciocalteu reagent, sodium carbonate, 1,1-diphenyl-2-picryl hydrazyl (DPPH) radical, potassium iodate (KIO_3_), bovine serum albumin (BSA), sodium nitrate (NaNO_3_), sodium dodecyl sulfate (SDS), triethanolamine, aluminum chloride (AlCl_3_), 6-hydroxy-2,5,7,8-tetramethylchroman-2-carboxylic acid (Trolox), gallic acid, quercetin, and tannic acid were purchased from Sigma-Aldrich S.p.a. (Milan, Italy).

### 2.2. Wood Samples

A total of 5 kg of Turkey oak chips (*Quercus cerris* L.) were used for the experimental analyses, taken randomly from a stock of 100 kg material from the sawmill line of Meridiana Legnami s.r.l (Tito scalo, Basilicata Region, Italy). The wood came from sawing residues of freshly processed Lucanian Turkey oak trunks with a moisture content of approximately 55.5%, and was measured using the gravimetric method. Moisture content measurements were carried out according to UNI ISO 1985 [17]. Half of the material was kept in the laboratory at room temperature (20 °C and 65% RH) as a control, while the other half was selected to obtain a more homogeneous substrate in terms of size which was without bark.

### 2.3. Experimental Design

The use of a Full Factorial Design (FFD) is an effective way to study the combined effects of multiple factors on one or more responses [18]. In the case of the present investigation, the extraction process was designed considering, as a starting point, a previous study performed on *Q. cerris* using microwave extraction and pure water [12]. Considering that microwave extraction is not always applicable on a large industrial scale, in this study, it was decided to employ extraction methods such as maceration and digestion (50 and 80 °C) in pure water or a hydroalcoholic mixture (20 and 40% of ethanol). Specifically, the three-level FFD [18] was used to optimize the extraction variables and maximize the content of specialized metabolites recovered from wood chips of *Q. cerris*. Thus, three independent variables, temperature (X_1_: 25, 50, and 80 °C), solvent (X_2_: 0, 20, 40% EtOH/H_2_O), and extraction time (X_3_: 3, 6, 24 h), were selected for a total of 27 empirical experiments (Table 1).

Different assays, such as DPPH scavenging activity, FRAP, TPC, TFC, CTC, and HTC, were used as dependent variables to analyze the response in terms of specialized metabolites and antioxidant activity. 

A linear regression equation was used to predict the optimal conditions and analyze the experimental data:

Y_n_ = *b*_0_ + *b*_1_X_1_ + *b*_2_X_2_ + *b*_3_X_3_ + *b*_12_X_1_X_2_ + *b*_13_X_1_X_3_ + *b*_23_X_2_X_3_ + *b*_123_X_1_X_2_X_3_

Y_n_ indicates the responses that were the radical-scavenging activity in mg TE/g (Y_DPPH_), ferric reducing antioxidant power in mg TE/g (Y_FRAP_), phenolic molecules in mg GAE/g (Y_TPC_), total content of flavonoids in mg TE/g (Y_TFC_), condensed tannins content (Y_TCT_), and hydrolyzable tannins content in mg TAE/g (Y_HTC_). X_1_, X_2_, X_3_ indicate the independent variables (temperature, solvent, and time), while *b*_0_ represents the constant regression coefficient; *b*_1_, *b*_2_, *b*_3_ are the linear regression coefficient; *b*_12_, *b*_13_ *b*_23_ are the regression coefficient of two-factor interaction; and *b*_123_ is the regression coefficient of three-factor interaction. The regression coefficients of the FFD model were calculated using MINITAB software package (Minitab^®^ Version 19.2020.1, 64-bit; LLC, State College, PA, USA), while the model’s adequacy and the statistical significative parameters were determined using the Fisher’s test for analysis of variance (ANOVA).

### 2.4. Extraction Procedure and Yield

For all extractions, 10 g of small-sized wood (20 × 15 × 3 mm) was extracted using the abovementioned parameters (Section 2.3). A constant solid–solvent ratio of 1:10 (*w*/*v*) was maintained during the experiments [19]. Extractions were carried out in a thermostatically controlled water bath associated with a horizontal forward/reverse type agitation with a 15 mm stroke (Memmert shaking water bath). For all extractions, agitation was maintained at 150 strokes per minute. After the extraction, the solutions were filtered, and the solvent was removed with a rotary evaporator at 37 °C. Dried extracts were kept in the dark at room temperature until their use. Extraction yields were calculated according to the following formula:

$$\%=\frac{dried\;extracts\;(g)}{milled\;wood\;(g)}\cdot 100$$

### 2.5. 2,2-Diphenyl-1-Picrylhydrazyl (DPPH) Scavenging Activity Assay

The DPPH scavenging activity assay is a commonly employed method to evaluate the antioxidant activity of specialized molecules and plant extracts. DPPH, a stable free radical, interacts with antioxidants, resulting in a color shift from purple to yellow that can be measured using a spectrophotometer. This color change signifies that DPPH has accepted an electron or hydrogen radical from the antioxidant, reflecting its scavenging or neutralizing capacity. In summary, 50 μL of various dilutions of Trolox or extract were mixed in a 96-well plate with 200 μL of DPPH methanol solution (100 μM). After 30 min of incubation in the dark at room temperature, absorbance was measured at 515 nm. A reduction in the absorbance of the DPPH solution corresponds to an increase in DPPH radical scavenging activity. The results were reported as milligrams of Trolox Equivalent (mg TE) per gram of dried extract. Each reaction was conducted in triplicate [20].

### 2.6. Ferric Reducing Antioxidant Power (FRAP) Assay

The FRAP assay evaluates the antioxidant activity of extracts by measuring the reduction of ferric iron (Fe^3+^) to ferrous iron (Fe^2+^) by antioxidant molecules. In brief, 25 μL of extract or Trolox was added to 225 μL of FRAP reagent. The FRAP reagent consisted of a mixture of 300 mM acetate buffer (pH 3.6), 20 mM FeCl_3_∙6H_2_O in distilled water, and 10 mM tripyridyltriazine (TPTZ) in 40 mM HCl, combined in a 10:1:1 ratio. This mixture was incubated at 37 °C for 40 min in the dark. Absorbance was then measured at 593 nm. An increase in the absorbance of the reaction mixture indicates a higher reduction capacity. Each reaction was performed in triplicate. Trolox served as the standard, and results were expressed as milligrams of Trolox equivalent per gram of dried extract (mg TE/g) [20].

### 2.7. Measurement of Total Phenol Content (TPC)

The total polyphenol content (TPC) was assessed using the Folin–Ciocalteu method [20]. In this procedure, 75 μL of extract was combined with 500 μL of Folin–Ciocalteu reagent and 500 μL of a 10× Na_2_CO_3_ solution (10 g of Na_2_CO_3_ in 100 mL of H_2_O). Water (425 μL) was then added to achieve a final volume of 1.5 mL. The mixture was incubated in the dark at room temperature for 1 h, after which the absorbance was measured at 723 nm using a spectrophotometer. The experiment was performed in triplicate, and results were reported as milligrams of gallic acid equivalent (GAE) per gram of dried extract (mg GAE/g).

### 2.8. Measurement of Total Flavonoid Content (TFC)

The total flavonoid content (TFC) was measured using an aluminum chloride assay [20], with quercetin serving as the standard reference. In this method, 500 μL of extract was mixed with 15 μL of 5× NaNO_3_ (5 g of NaNO_3_ in 100 mL of H_2_O). After 5 min, 30 μL of 10× AlCl_3_ (10 g of AlCl_3_ in 100 mL of H_2_O) was added. Following 1 min, 100 μL of 1 mM NaOH was added to the reaction mixture, which was then diluted with 255 μL of water. The absorbance of the mixture was recorded at 510 nm using a spectrophotometer. The experiment was conducted in triplicate, and the results were reported as milligrams of quercetin equivalents (QE) per gram of dried extract (mg QE/g).

### 2.9. Measurement of Condensed Tannin Content (CTC)

The condensed tannin content (CTC) was assessed through precipitation with bovine serum albumin (BSA), following the method described by Libutti et al. [20]. In this procedure, 250 μL of extract was mixed with 500 μL of 0.2 M BSA in acetate buffer (pH 5.0, containing 0.17 M NaCl). After a 15 min incubation period, the mixture was centrifuged at 5000 rpm for 15 min, and the supernatant was discarded. The precipitate was then diluted with 1 mL of a solution containing 1% SDS, 4% triethanolamine, and 250 μL of 0.01 M FeCl_3_ in 0.01 M HCl. After a 30 min incubation period, absorbance was measured at 510 nm using a spectrophotometer. The experiment was conducted in triplicate, and the results were reported as milligrams of tannic acid equivalent (TAE) per gram of dried extract (mg TAE/g), with the standard deviation (SD) provided.

### 2.10. Measurement of Hydrolyzable Tannin Content (HTC)

The hydrolyzable tannin content (HTC) was determined using a modified version of the method by Libutti et al. [20]. In this assay, 50 μL of extract was mixed with 200 μL of 2.5% (*w*/*v*) KIO_3_ solution. The mixture was incubated for 7 min at 30 °C, and then its absorbance was measured at 550 nm using a spectrophotometer. A calibration curve was prepared using tannic acid. The experiment was performed in triplicate, and the results were expressed as milligrams of tannic acid equivalents (TAE) per gram of dried extract (mg TAE/g), along with the standard deviation (SD).

### 2.11. Artificial Neural Network (ANN) Modeling

**Figure 1:**
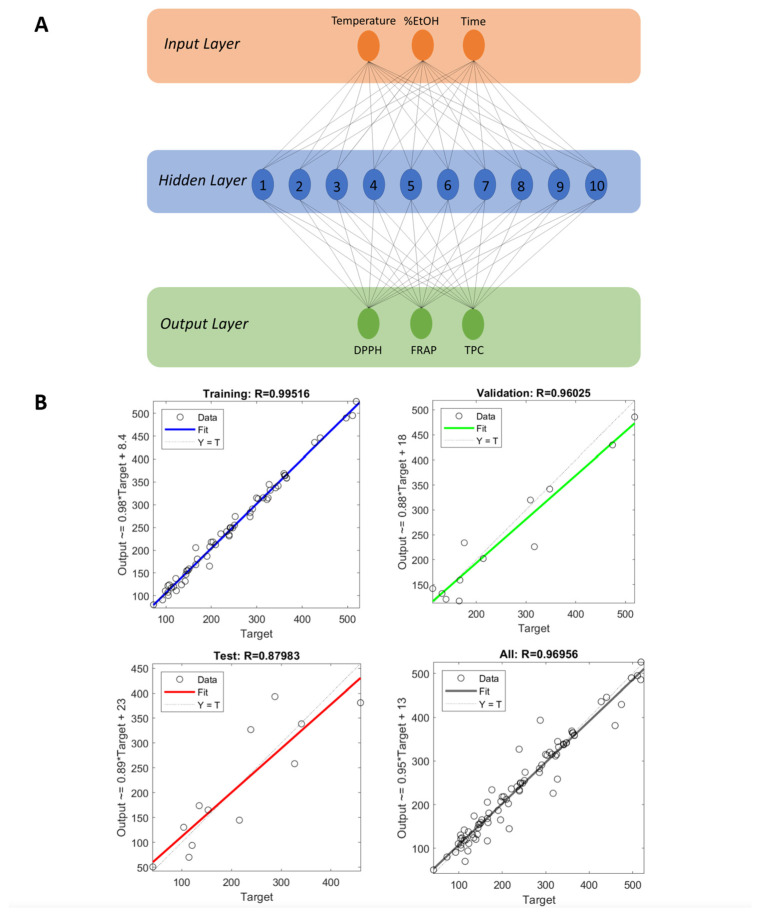
(**A**) General architecture of the feed-forward backpropagation multilayer perceptron (MLP) neural network consisting of 3 neurons as the input layer, 10 as the hidden layer, and 3 as the output layer. (**B**) Training, validation, testing, and overall datasets’ correlation coefficients (R) for the developed ANN model.

The neural network-fitting tool from MATLAB R2023b (The MathWorks Inc., Natick, MA, USA) was used to model experimental data generated from the extraction of Turkey oak chips. Multilayer perceptron (MLP) is a widely employed technique within ANN for data modeling. MLP consists of a feed-forward (FF) backpropagation (BP) algorithm, incorporating input layers, hidden layers, and output layers (Figure 1).

Each layer may contain one or multiple neurons. ANN model performance is strongly related to the number of neurons in the hidden layer, since a very small amount of neutrons may reduce the ANN’s ability to model the process appropriately, and the network can be poorly trained, whereas too many hidden layer neurons can cause the network to store data instead of training. In the present case, the number of hidden layers varied between 8 and 12 to train the ANN (Figure S1). A sigmoid transfer function was used between the input layer and the hidden layer, as well as between the hidden layer and the output layer. The ANN model was trained until the error between the experimental values and the predicted values of the responses reached the minimum value. The Levenberg–Marquardt training algorithm was employed to train the data set. The trained model was validated employing a validation data set comprising experimental data not included in the training. Thus, the ANN model was developed by dividing the dataset into three groups: 70% for training (19 observations), 15% for testing (4 observations), and 15% for validating (4 observations). The ANN model training was performed until the minimum mean squared error (MSE) was reached in the validation process.

### 2.12. Comparison of FFD and ANN Models Prediction Ability

For comparing the FFD and ANN models’ estimation capabilities, the coefficient of determination (R^2^), mean absolute error (MAE), and root mean square error (RMSE) were calculated using the following equations:

$$R^{2}=\;1-\frac{\sum_{i=1}^{n}(Y_{pre}-Y_{exp}{)}^{2}}{\sum_{i=1}^{n}(Y_{m}-Y_{exp}{)}^{2}}$$

$$\mathrm{RMSE}=\sqrt{\frac{\sum_{i=1}^{n}(Y_{pre}-Y_{exp}{)}^{2}}{n}}$$

$$\mathrm{MAE}=\;\frac{1}{n}\sum_{i=1}^{n}\left|Y_{pre}-\left.Y_{exp}\right|\right.$$

where *n* is the data point number, $Y_{pre}$ is the FDD or ANN predicted response variable, $Y_{exp}$ is the experimental variable, and $Y_{m}$ is the response variable average. 

### 2.13. Optimization of the Process

FFD optimization process was performed using the response optimizer function implemented in MINITAB software package (Minitab^®^ Version 19.2020.1, 64-bit; LLC, State College, PA, USA) by maximizing the analyzed responses. On the other hand, the optimization of the trained ANN model was performed using the numerical optimization option implemented in DesignExpert13 (version 13, Design-Expert Software, Stat-Ease Inc., Minneapolis, MN, USA) by inserting the ANN-predicted data for DPPH scavenging activity, FRAP, and TPC as responses. 

### 2.14. Statistical Analysis

Results were expressed as the mean ± standard deviation (SD) of three independent experiments performed in triplicate. Statistical analysis was performed with a one-way analysis of variance (ANOVA). The Minitab17 statistical software was used to create the experimental design and analyze the experimental data. Differences between groups were determined using the Tukey test; differences were considered statistically significant when *p* < 0.05. A Pearson correlation analysis was performed to determine the relationship among all selected variables.

## 3. Results and Discussion

### 3.1. Model Adequacy

This investigation aimed to construct an FFD for obtaining extracts from *Q. cerris* wood chips with the highest antioxidant activity, assessed through DPPH scavenging activity and FRAP assays, as well as the highest content of specialized metabolites, measured using TPC, TFC, CTC, and HTC assays. The FFD was modeled on three levels (cf. Table 1) assigned to each factor. The ANOVA test was used to estimate the effect of the variables analyzed on the response, their interactions, and the model’s statistical significance. All results obtained from the in vitro tests with the corresponding statistical analyses are presented in Table 2, while data from the ANOVA analysis are reported in Table S1.

In all cases, no data transformation (e.g., Square roots, Natural Log, Base 10 Log, etc.) was applied. The statistically significant *p*-values of the linear model suggest that the developed model provided an acceptable estimation for the tested responses (Table S1). However, if Fit statistics were evaluated, it was possible to observe that, for CTC, the built model was not suitable (Table 3). In contrast, for DPPH scavenging activity, FRAP, TPC, TFC, and HTC, the good fit between the predicted and experimental results was confirmed by the regression coefficient (R^2^) values and by the difference between Adj. R^2^ and Pred. R^2^, since in all cases, it was less than 0.2. Also noteworthy is the Adequate Precision measuring the signal-to-noise ratio. Generally, a ratio greater than 4 is desirable; for DPPH scavenging activity, FRAP, TPC, TFC, and HTC, the Adequate Precision was between 9.1674 and 14.2945, indicating that the model can be used to navigate the design space.

**Figure 2:**
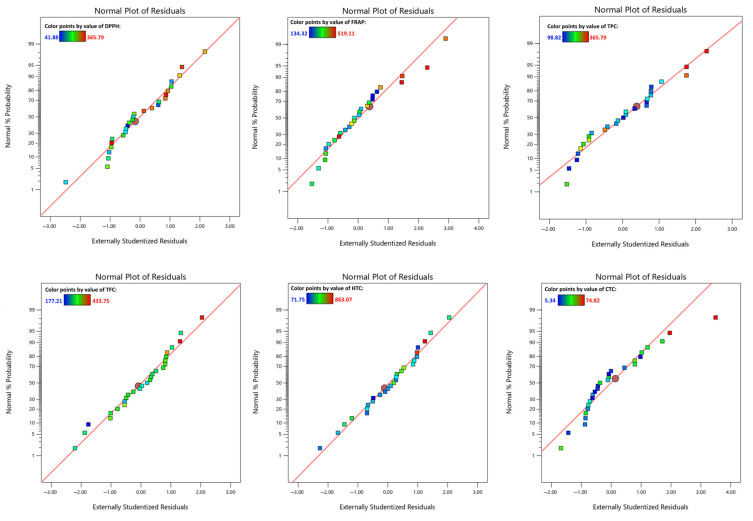
Residual analysis of the model.

In addition, to understand whether the model residuals followed a normal distribution, normal residuals plots were evaluated. As shown in Figure 2, most of the residuals evaluated for each response exhibited a normal distribution, i.e., close to the normal distribution (red line). The only exception was visible for the normal residuals plot of the CTC, where there were more obvious deviations, suggesting the possibility of outliers or residuals not normally distributed.

Based on the data obtained, the optimization analysis was continued by considering DPPH scavenging activity, FRAP, TPC, TFC, and HTC parameters. The linear regression equations for all factors and interactions were used to make predictions about the response for the given levels of each factor, thus identifying the impact of the independent variables on the response (Table 4).

### 3.2. Effect of Extraction Parameters on Yield

Generally, the properties and yield of secondary metabolites from natural sources are strongly associated with various parameters, such as extraction technique, solvent, temperature, and extraction time [21]. Temperature is one of the fundamental variables to consider when recovering secondary metabolites from plant matrices. As widely reported by Rakić et al. (2007) [13], increasing the temperature increases the extraction yield. The type of plant matrix to be sought determines the critical temperature selection during the experimental design phase. In the case of the present work, the highest yield was obtained at a temperature of 80 °C and this yield further increased as the extraction time increased (3 h, 6 h, 24 h), in line with the results obtained by Gironi and Piemonte [22]. Furthermore, a significant increase in yield was observed as the percentage of ethanol used increased; in fact, the 40% ethanol made it possible to obtain the highest extraction yield. Ethanol is certainly among the most used organic solvents due to its low environmental impact and lower harmfulness to human health than other organic solvents. As can be seen in Table 2, the concentration of ethanol, temperature, and extraction time positively influence the extraction yield (ranging from 0.61% to 2.85%). The greatest yields were obtained by performing the decoction with the highest concentration of ethanol (EtOH: H_2_O, 40:60 *v*/*v*), at the maximum temperature (80 °C) and by increasing the extraction time (24 h). To investigate the correlation between yield and the evaluated responses, Pearson correlation was calculated (Table S2), which showed that it was mainly correlated with TPC and TFC (0.811 and 0.787, respectively) and, consequently, with the antioxidant activity evaluated by FRAP and DPPH scavenging activity assay (0.779 and 0.791, respectively).

### 3.3. Effect of Extraction Conditions on the Antioxidant Activity Based FFD

Antioxidant activity was measured through two spectrophotometric complementary tests, DPPH scavenging activity and FRAP assay. As far as is known, this is the first investigation evaluating the antioxidant activity of *Q. cerris* wood chips. For instance, Gortzi, et al. [23] examined the antioxidant activity and sensory properties of two Greek red wines artificially aged using different wood chips. Nisca et al. (2022) [12] investigated the radical scavenging activity of aqueous and hydroalcoholic extracts of *Q. cerris* bark, achieving an IC_50_ ranging from 2.44 ± 0.24 to 6.92 ± 0.39 using pyrogallol as standard (IC_50_ 6.67 ± 0.47). In the present investigation, the ranges of milligrams of Trolox equivalents per gram of dry extract (DW) were from 41.88 ± 7.46 to 365.78 ± 1.95 and 134.32 ± 9.15 to 519.11 ± 41.19 for DPPH scavenging activity and FRAP, respectively. The results obtained were slightly lower than those of Sen, et al. [24], which, for the antioxidant activity evaluated with the DPPH scavenging activity assay, obtained values between 248.40 ± 0.10 and 1151.4 ± 18.79 mgTE/g. However, it is necessary to consider that they analyzed the cork-rich fractions and phloem extracts. Hence, the observed discrepancy may be related to the sample material (bark) and different sample preparations, since the trituration process with the hammer mill was used before the extraction [24].

**Figure 3:**
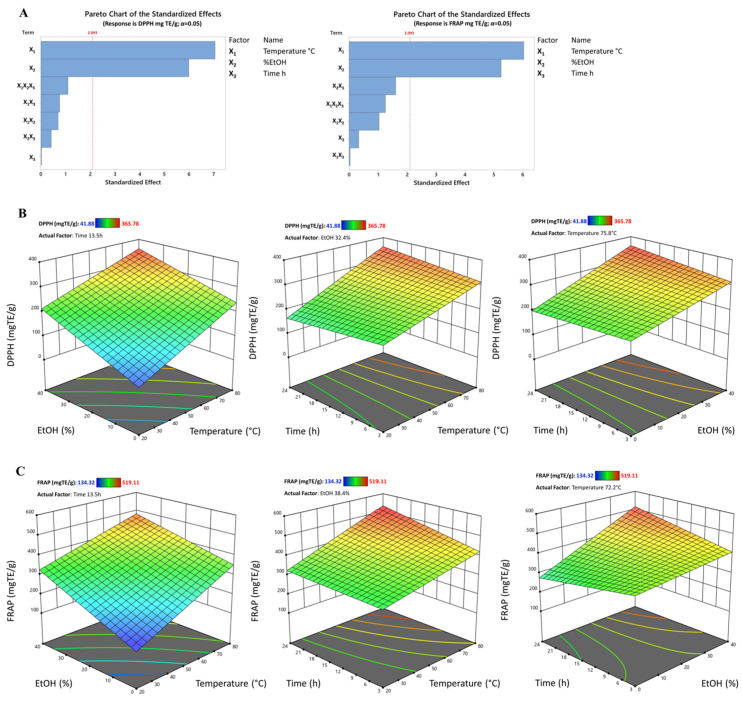
(**A**) Effects of independent variables (time, temperature, and solvent) on antioxidant activity; surface and contour plot of (**B**) 1,1-diphenyl-2-picryl hydrazyl (DPPH) scavenging activity and (**C**) Ferric Reducing Antioxidant Power (FRAP).

The analysis of variance (ANOVA) was applied to identify the parameters that most influenced the extraction of metabolites conferring antioxidant activity to *Q. cerris* wood chip extract. Specifically, as was possible to observe in the surface plots and Pareto charts of the standardized effects, including the impact of the parameters (level of significance *α* = 0.05), temperature and EtOH concentration were the parameters that most influenced the antioxidant activity (Figure 3).

Hence, the optimal extractive solutions capable of yielding extracts with the maximum antioxidant activity in terms of mg TE/g were evaluated for either DPPH scavenging activity or FRAP assays. Particularly, the optimal extraction conditions for maximizing DPPH scavenging activity were X_1_ = 79.986 °C, X_2_ = 39.388% EtOH/H_2_O, and X_3_ = 16.281 h, which allowed 365.925 mg TE/g (composite desirability = 1.000) to be obtained. On the other hand, the maximization of the FRAP values (519.177 mg TE/g; composite desirability = 1.000) was achieved using the combination of the following parameters: X_1_ = 79.942 °C, X_2_ = 39.913% EtOH/H_2_O, and X_3_ = 19.894 h. As it was possible to observe, the predicted extraction conditions confirmed that the extraction time did not contribute to the increase in antioxidant activity, since the maximum value were applied only for temperature and percentage of EtOH. Previous investigations indeed reported that polyphenol degradation may occur with longer extraction times [25]. On the other hand, it was demonstrated that increasing the ethanol concentration and temperature plays a pivotal role in extracting antioxidant compounds [9].

### 3.4. Effect of Extraction Conditions on Specialized Metabolites Based FFD

Several studies have identified phenolic compounds from *Quercus* spp. which are known for their healthy properties, making them useful in nutraceutical fields, such as catechin, gallic acid, vanillic acid, syringaldehyde, caffeic acid, ferulic acid, ellagic acid, and others [3]. Therefore, to optimize the contents of specialized metabolites extracted from wood chips of *Q. cerris*, all extracts were tested for the total phenolic content (TPC), flavonoids (TFC), and hydrolyzable tannins (HTC).

#### 3.4.1. Effect on Total Phenolic Content (TPC)

**Figure 4:**
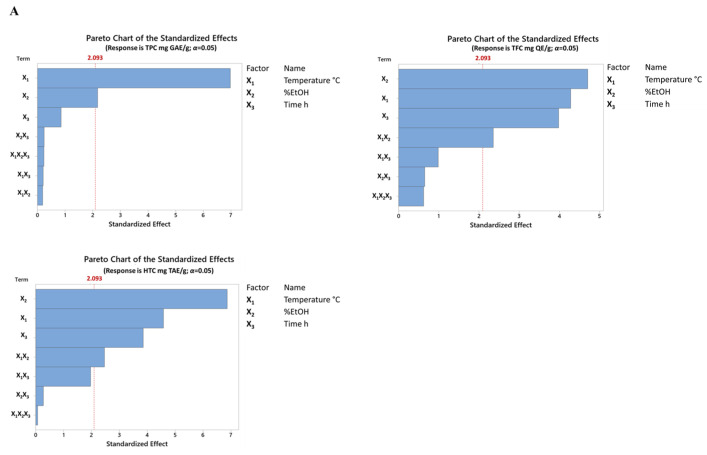

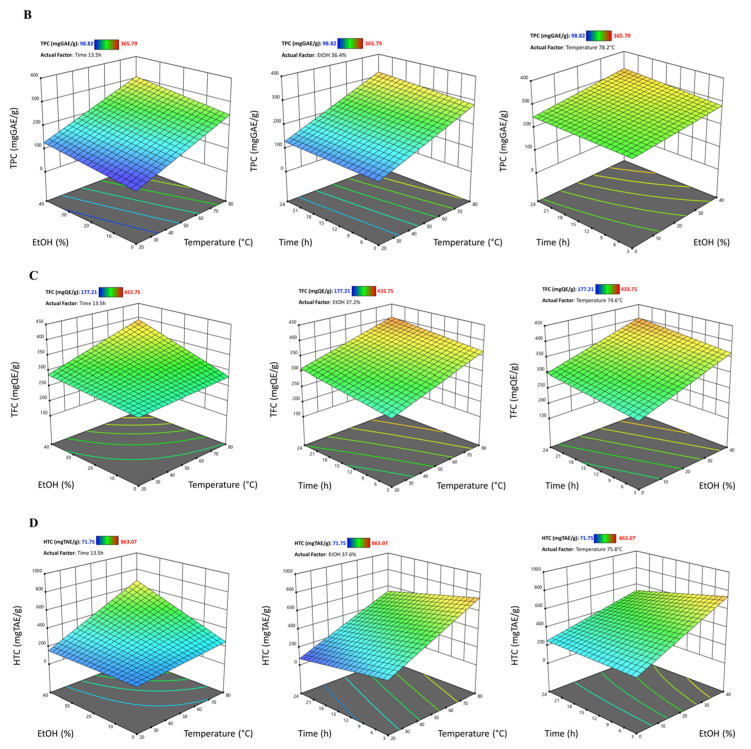
(**A**) Effects of independent variables (time, temperature, and solvent) on extraction of specialized metabolites. Surface and contour plot of (**B**) total phenolic content (TPC), (**C**) total flavonoid content (TFC), and (**D**) hydrolyzable tannin content (HTC).

Concerning the total phenolic compound content, Nisca et al. (2022) reported that the TPC ranged from 284.2 to 338.8 and 284.2 to 338.8 mg GAE/g using microwave-assisted extraction (MAE) and an ultrasonic bath, respectively [12]. Additionally, Stefanescu et al. confirmed the highest value of the TPC (347.74 mg/g) obtained using MAE and a hydroethanolic mixture [26]. Similarly, in this work, using maceration and decoction, the polyphenol content in the analyzed extracts ranged between 98.82 ± 9.49 and 365.79 ± 14.77 mg GAE/g of dry extract. These results align with the content found by Ferreira, et al. [27] in the cork back and cork ethanol extracts, with a range from 189.9 ± 19.6 to 435.1 ± 8.1 mg GAE/g DW. Although the sample materials are different in many manuscripts, the amount of TPC obtained from wood chips of *Q. cerris* by applying maceration and decoction is comparable to advanced extraction methods such as MAE and UAE, as mentioned before. Specifically, a temperature of 80 °C allowed a higher phenolic content to be obtained than that obtained at lower temperatures of 20 °C and 40 °C. In addition, although to a lesser extent than temperature, the percentage of ethanol also influenced the extraction of phenolic compounds (Figure 4A,B).

Based on the data obtained from the linear regression model, the optimal extraction conditions for maximizing the total phenols were estimated to be X_1_ = 80 °C, X_2_ = 40% EtOH/H_2_O, and X_3_ = 23.62 h, with a predicted phenolic concentration of 326.094 mg GAE/g (composite desirability = 0.851).

#### 3.4.2. Effect on Total Flavonoid Content (TFC)

Table 1 shows the amount of TFC in all extracts carried out by applying the experimental model. Bajraktari et al. investigated the presence of flavonoids in Turkey oak wood ethanol extracts harvested in the Republic of Kosovo, displaying results expressed in mg of catechin equivalent per gram of dry extract (mg CE/g DW) with a range from 61.2 to 67.4 [28]. By contrast, Sen et al. obtained a range from 101.92 to 460.91 mg CE/g DW. Although the same solvent combination (50% EtOH/H_2_O) was employed, differences in the contents of flavonoids may be related to the starting material used (cork-rich, phloem-rich, or heartwood) and from the environmental and climatic conditions in which the species grew. In fact, in previous studies, the flavonoid content of wood extracts as starting materials ranged from 85.0 ± 3.0 to 263.4 ± 4.6 mg QE/g. [24,29]. However, as no other studies have been conducted on wood chips of *Q. cerris*, it is not possible to make a direct comparison of the flavonoid contents obtained in the present investigation, which ranged between 177.21 ± 10.91 and 433.75 ± 15.57 mg QE/g of dry extract (cf. Table 1).

Regarding the analysis of the parameters, as can be observed in the Pareto chart and linear regression equation (Figure 4A–C and cf. Table 4), it was evident that all the extraction conditions (temperature, percentage of ethanol, and time) affected the extraction of flavonoids. Based on the optimization analyses, the optimal extraction parameters that might maximize the total flavonoid content were: X_1_ = 80 °C, X_2_ = 40% EtOH/H_2_O, and X_3_ = 24 h, with a predicted concentration of 412.1 mg GAE/g (composite desirability = 0.916).

#### 3.4.3. Effect Hydrolyzable Tannin Content (HTC)

Tannins are polyphenolic compounds, soluble in water, and often associated with woody tissues. Based on their structure and stability, these metabolites can be classified as hydrolyzable and condensed [30]. As reported in Table 1, the levels of HTC ranged between 71.75 ± 0.96 and 863.07 ± 25.67 mg TAE/g. In contrast, in another investigation, hydrolyzable tannins were not found in ethanol–water extracts maintained at 40 °C in an ultrasonic bath [28]. It can be assumed that the observed difference in the hydrolyzed samples may be due to divergent sample preparation, maturity stage, collection area, and expression data.

As depicted in Figure 4A–D and cf. Table 4, the parameters exerting the greatest influence on the extraction of hydrolyzable tannins were temperature, percentage of ethanol, and extraction time. However, the minus sign preceding the time value in the equation (cf. Table 4) indicates that the HTC concentration was inversely proportional to time. In fact, by determining the optimal extraction conditions, it can be seen that while maximum values were applied for the temperature and ethanol percentages, the minimum value was applied for time: X_1_ = 80 °C, X_2_ = 40% EtOH/H_2_O, and X_3_ = 3 h, with a predicted concentration of 776.929 mg TAE/g (composite desirability = 0.891).

### 3.5. Multiple Response Prediction

The ultimate goal of the optimization process is to identify the combination of conditions that would yield extracts with the highest specialized metabolite concentrations. Following the analysis of the results, it was concluded that a temperature of 80 °C, an ethanol concentration of 40%, and an extraction duration of 19.8 h represented the optimal conditions for achieving the maximum levels of all desired outcomes (Table 5).

In Table 5, the value of SE Fit indicates how accurate the model is in predicting each response value. Lower values indicate more accurate predictions; in this case, the best prediction was obtained for TFC, followed by DPPH scavenging activity, TPC, FRAP, and HTC.

### 3.6. Pearson Correlation Existing between the Dependent Variables

The Pearson correlation method is used to observe the correlations between all dependent variables used to perform the FFD. In statistics, the Pearson correlation index between two statistical variables is an index that expresses a possible linear relationship between them. In line with the capacity to evaluate antioxidant activity, data from the FRAP assay are strongly related to the DPPH scavenging activity test, with a high R^2^ = 0.931. As is known, the antioxidant efficacy of phenolic compounds stems from their ability to donate hydrogen or electrons, indicating their potential to function as scavengers of free radicals. This is further confirmed by the correlation of TPC with both DPPH scavenging activity and FRAP assays (R^2^ = 0.737 and R^2^ = 0.733 for DPPH scavenging activity and FRAP assay, respectively). On the other hand, regarding the class of phenols investigated, the higher correlation with the antioxidant activity is related to condensed tannins (R^2^ = 0.706 and R^2^ = 0.748 for DPPH scavenging activity and FRAP assay, respectively), followed by hydrolyzed tannins and flavonoids (Table 6).

### 3.7. Artificial Neural Network Model

Considering that the Pearson analysis found a correlation between FRAP, DPPH scavenging activity, and TPC, and taking into account the ability of TPC to provide a measure of the total reducing power of samples [31], the ANN model was built based on these three variables indicating the antioxidant activity of the *Q. cerris* extracts. The ANN network architecture is reported in Figure 1. The feed-forward back-propagation model with three layers, i.e., an input layer, a hidden layer, and an output layer, was used for the ANN training, testing, and validation. The input and output layers contained three neurons corresponding to the independent and selected dependent variables, respectively. In the hidden layers, the number of neurons was changed from 8 to 12 (Figure S1). ANN’s best topology (hidden layer neuron numbers), corresponding to the highest R^2^, was found when the neuron number was set at 10 (cf. Figure 1A). Hence, the final topology consisted of three layers for input and output layers and ten neurons for the hidden layer (Figure 1A). The R^2^ correlation coefficients for training, testing, and validation were 0.995, 0.960, and 0.880, respectively, indicating that the developed model was sufficiently reliable in predicting outputs for different input sets (cf. Figure 1B). The sizes of the weight matrices connecting the input layer neurons to the hidden layer were 10 × 3, and the weight matrices connecting the hidden layer to the output layer were 3 × 10, while the sizes of the hidden layer bias matrices were 10 × 1 and the output layer bias matrix was 3 × 1.

**Figure 5:**
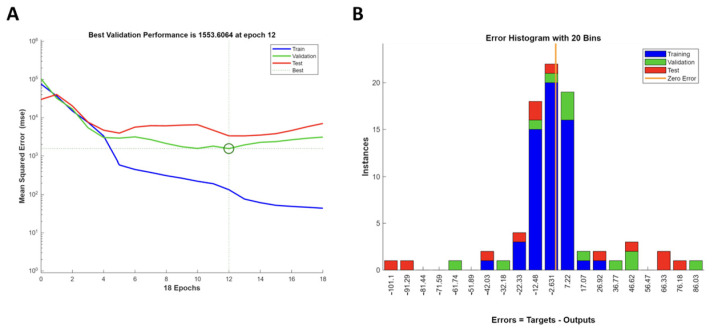
Artificial neural network model validation. (**A**) Best validation performance plot. (**B**) Error histogram.

As shown in Figure 5A, the training error continuously decreased as the number of epochs increased.

This is a typical behavior as the model improves using the training data. Initially, the validation error decreases along with the training error, but around epoch 12, it stops improving and begins to rise again. This indicates that after epoch 12, the model starts overfitting the training data because it is performing well on the training data but starts to generalize to the validation data poorly. The test error shows a similar pattern to the validation error, starting to increase around the same epoch where overfitting begins. This further confirms that the model is overfitting after a certain point. For this reason, the training was stopped at epoch 12. To further validate the model, the error histogram showing the distribution of errors (differences between the targets and the outputs) for the training, validation, and test sets was considered. As can be observed in Figure 5B, the model fit the data reasonably well, as the majority of errors were near zero, especially for the training set.

The weights and biases of the developed ANN model are shown in the Supplementary Material (Table S3).

### 3.8. Comparison of FFD and ANN Models

**Figure 6:**
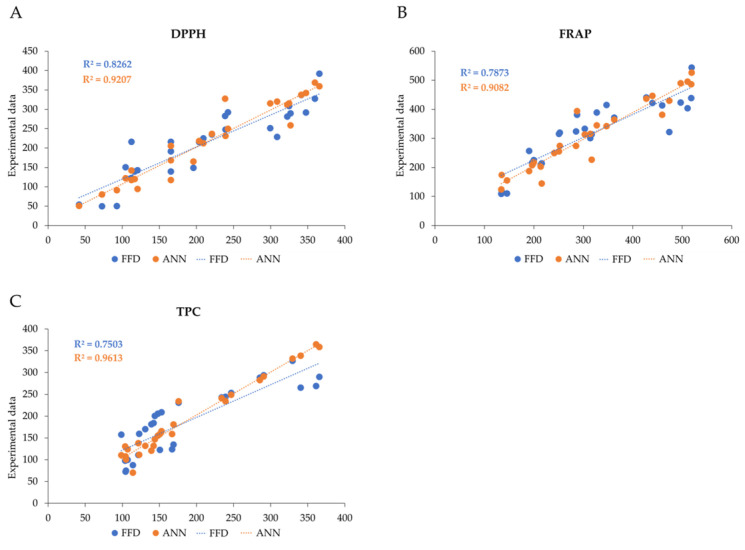
Performance comparison of ANN (in orange) and FFD (in blue) models for (**A**) DPPH: 2,2-diphenyl-1-picrylhydrazyl, expressed as milligrams of Trolox equivalents per gram of dry extract; (**B**) FRAP: ferric reducing antioxidant power, expressed as milligrams of Trolox equivalent per grams of dry extract; and (**C**) TPC: total phenolic content, expressed as milligrams of gallic acid per grams of dry extract.

FFD and ANN models were compared based on different variables, such as the RMSE, the MAE, and R^2^ (Figure 6).

As can be observed in Table 7, the FFD model resulted in a higher deviation than the ANN model, indicating that the ANN model, if well trained, results in the greatest predictive accuracy.

The ANN’s model superiority in predictive capability has also been confirmed by other articles [32,33]. This can be related to the nature of the two methods, since the FFD model is limited to a linear regression equation, while the ANN model can approximate the system nonlinearly. Moreover, ANN has the advantage over the FFD in calculating, in a single process, multiple responses, while the FFD must be run for all parameters to be predicted. Nevertheless, FFD’s structured nature makes it helpful in gaining direct insight into the system (for example, interactions among different components), and it is easier to use than ANN. Therefore, both processes should be used to optimize the extraction process, as they have different characteristics.

### 3.9. Process Optimization

Either FFD or ANN models were applied to optimize the extraction of Turkey oak chips based on antioxidant activity and phenolic content. The optimized parameters proposed for both models are reported in Table 8 and resulted in similar extraction conditions and DPPH, FRAP, and TPC values. Therefore, both processes can be useful in optimizing the extraction process.

## 4. Conclusions

The research carried out in this work provides an exhaustive overview of the new paradigm of the circular bioeconomy and highlights the need to implement the valorization of wood by-products as a source of high-value specialized metabolites. Within this scope, dynamic maceration and decoction with ethanol and water not only allow the costs of a possible industrial scale-up process to be contained, but also have a low environmental impact, as they fall within the scope of the green economy if compared with other organic solvents and extraction methods like Soxhlet, UAE, or MAE, which may be more expensive. Applying the full factorial design (FFD), the optimization of the extraction process for the recovery of secondary metabolites from Turkey oak chips was carried out through the study of three different variables, i.e., ethanol concentration, temperature, and duration of the extraction process, keeping the drug–solvent ratio (1:10 g/mL) and the wood chips’ size constant. FFD was performed to maximize the TPC, TFC, and HTC, and the extracts’ antioxidant activity was evaluated by DPPH scavenging activity and a FRAP assay, obtaining the following optimal extraction conditions: temperature of 80 °C, percentage of ethanol in water of 40%, and extraction time of 19.8 h. Furthermore, the predictive capabilities of the FFD and ANN models were compared using the three dependent variables with the highest Pearson correlation: DPPH scavenging activity, FRAP, and TPC. Prediction evaluated with ANN was more accurate than that performed with FFD, resulting in higher R^2^ and lower MAE and RMSE values. However, the extraction optimization for DPPH scavenging activity, FRAP, and TPC, performed with the ANN and FFD models, resulted in similar values, indicating that both models can be suitable for this scope. Hence, this work laid the basis for valorizing, using different optimization models, wood chips of *Q. cerris*, which are currently deemed as industry waste even if they are rich in bioactive substances applicable in the nutraceutical field. Future studies will be conducted to experimentally validate the developed models and better elucidate the phytochemical characterization of the optimized extract.

## Figures and Tables

**Table 1:** Extraction variable parameters and experimental design.

	Symbols	Coded Levels		
Independent variables		−1	0	1
Temperature (°C)	X_1_	25	50	80
Solvent (% EtOH/H_2_O)	X_2_	0	20	40
Time (h)	X_3_	3	6	24

**Table 2:** Independent variables with the respective codes used in the FFD and results from in vitro assays.

	Independent Variables			Dependent Variables						
Run	X_1_	X_2_	X_3_	Yield%	^1^ DPPH Scavenging Activity mg TE/g DW	^2^ FRAP mg TE/g DW	^3^ TPC mg GAE/g DW	^4^ TFC mg QE/g DW	^5^ CTC mg TAE/g DW	^6^ HTC mg TAE/g DW
1	20 (−1)	0 (−1)	3 (−1)	0.61 ± 0.03 ^m^	72.69 ± 3.57 ^h, i^	134.32 ± 9.15 ^j^	104.45 ± 1.16 ^g^	177.21 ± 10.91 ^e^	13.09 ± 1.26 ^g, h, i, j^	161.82 ± 7.55 ^j, k^
2	20 (−1)	0 (−1)	6 (0)	0.63 ± 0.01 ^m^	92.66 ± 3.55 ^h^	145.65 ± 7.79 ^j^	105.18 ± 17.61 ^g^	234.19 ± 20.83 ^d, e^	5.34 ± 0.46 ^j^	187.64 ± 9.32 ^i, j, k^
3	20 (−1)	0 (−1)	24 (1)	0.78 ± 0.05 ^l, m^	41.88 ± 7.46 ^j^	135.10 ± 4.13 ^j^	114.46 ± 13.45 ^f, g^	299.03 ± 37.81 ^c, d, e^	5.70 ± 0.54 ^i, j^	202.88 ± 13.96 ^h, i, j^
4	20 (−1)	20 (0)	3 (−1)	0.67 ± 0.02 ^l, m^	120.78 ± 2.96 ^h^	215.87 ± 8.01 ^h, i, j^	103.87 ± 1.39 ^g^	225.38 ± 10.75 ^d, e^	5.55 ± 0.63 ^j^	182.83 ± 17.77 ^i, j, k^
5	20 (−1)	20 (0)	6 (0)	0.92 ± 0.08 ^j, k, l, m^	116.87 ± 5.94 ^h^	196.71 ± 11.69 ^i, j^	107.29 ± 45.76 ^g^	251.13 ± 10.17 ^d, e^	7.71 ± 0.88 ^h, i, j^	191.70 ± 15.54 ^i, j, k^
6	20 (−1)	20 (0)	24 (1)	1.03 ± 0.08 ^i, j, k, l, m^	112.53 ± 6.47 ^h^	199.78 ± 19.26 ^i, j^	121.82 ± 3.01 ^e, f, g^	301.84 ± 23.50 ^c, d^	5.60 ± 0.46 ^j^	71.75 ± 0.96 ^l^
7	20 (−1)	40 (1)	3 (−1)	0.76 ± 0.02 ^l, m^	220.89 ± 8.12 ^d, e, f^	252.48 ± 15.78 ^f, g, h, i^	150.76 ± 1.04 ^e, f, g^	277.22 ± 17.31 ^c, d, e^	19.51 ± 1.71 ^f, g^	301.72 ± 3.85 ^d, e, f^
8	20 (−1)	40 (1)	6 (0)	1.09 ± 0.03 ^i, j, k, l, m^	308.96 ± 14.91 ^c^	474.09 ± 44.52 ^b^	167.19 ± 8.83 ^e, f^	280.49 ± 28.01 ^c, d, e^	36.95 ± 2.43 ^c, d^	284.59 ± 26.30 ^e, f, g^
9	20 (−1)	40 (1)	24 (1)	1.46 ± 0.09 ^e, f, g, h, i^	165.69 ± 8.43 ^g^	303.11 ± 26.73 ^e, f, g^	169.16 ± 26.57 ^e, f^	324.47 ± 12.30 ^a, b, c, d^	36.57 ± 3.43 ^c, d^	118.97 ± 10.74 ^k, l^
10	50 (0)	0 (−1)	3 (−1)	0.87 ± 0.07 ^k, l, m^	104.4 ± 6.85 ^h^	190.43 ± 6.28 ^i, j^	98.82 ± 9.49 ^g^	276.22 ± 23.26 ^c, d, e^	38.75 ± 3.64 ^b, c, d^	217.85 ± 10.30 ^g, h, i, j^
11	50 (0)	0 (−1)	6 (0)	1.03 ± 0.04 ^i, j, k, l, m^	195.99 ± 15.98 ^f^	241.46 ± 13.35 ^g, h, i^	122.95 ± 4.07 ^e, f, g^	278.36 ± 25.93 ^c, d, e^	8.74 ± 0.77 ^h, i, j^	371.73 ± 32.03 ^c, d^
12	50 (0)	0 (−1)	24 (1)	1.12 ± 0.08 ^i, j, k, l^	165.69 ± 8.43 _g_	214.05 ± 16.02 ^h, i, j^	131.17 ± 0.12 ^e, f, g^	323.66 ± 25.68 ^a, b, c, d^	8.97 ± 0.16 ^h, i, j^	152.46 ± 12.57 ^j, k^
13	50 (0)	20 (0)	3 (−1)	1.30 ± 0.05 ^h, i, j, k^	112.44 ± 6.05 ^h^	316.91 ± 17.35 ^e, f, g^	139.18 ± 13.21 ^e, f, g^	301.84 ± 11.23 ^c, d^	13.51 ± 4.33 ^g, h, i, j^	218.73 ± 8.00 ^g, h, i, j^
14	50 (0)	20 (0)	6 (0)	1.62 ± 0.07 ^e, f, g, h^	165.69 ± 8.43 ^g^	250.94 ± 21.47 ^f, g, h, i^	142.38 ± 0.37 ^e, f, g^	312.25 ± 27.83 ^a, b, c, d^	14.50 ± 1.33 ^g, h, i^	162.55 ± 14.71 ^j, k^
15	50 (0)	20 (0)	24 (1)	1.73 ± 0.08 ^e, f, g, h^	203.64 ± 5.75 ^f^	285.5 ± 11.90 ^e, f, g, h^	143.95 ± 4.09 ^e, f, g^	317.85 ± 31.11 ^a, b, c, d^	13.60 ± 0.48 ^g, h, i, j^	217.51 ± 20.11 ^g, h, i, j^
16	50 (0)	40 (1)	3 (−1)	1.44 ± 0.05 ^f, g, h, i^	322.10 ± 9.75 ^b, c^	362.79 ± 3.36 ^c, d, e^	148.16 ± 17.27 ^e, f, g^	297.84 ± 9.02 ^c, d, e^	46.79 ± 3.06 ^b^	410.08 ± 35.66 ^c^
17	50 (0)	40 (1)	6 (0)	1.69 ± 0.06 ^e, f, g, h^	238.77 ± 7.92 ^d, e^	287.31 ± 21.35 ^e, f, g, h^	152.97 ± 9.10 ^e, f, g^	297.04 ± 13.25 ^c, d, e^	26.74 ± 2.55 ^e, f^	521.61 ± 45.63 ^b^
18	50 (0)	40 (1)	24 (1)	2.33 ± 0.14 ^b, c^	347.71 ± 25.97 ^a, b^	518.48 ± 51.93 ^a^	175.98 ± 17.70 ^d, e^	347.08 ± 24.71 ^a, b, c, d^	38.18 ± 2.98 ^b, c, d^	261.22 ± 23.77 ^f, g, h, i^
19	80 (1)	0 (−1)	3 (−1)	1.39 ± 0.08 ^g, h, i, j^	299.60 ± 5.66 ^c^	511.04 ± 26.71 ^a, b^	234.04 ± 6.43 ^c, d^	263.61 ± 16.89 ^d, e^	34.10 ± 1.48 ^d, e^	203.48 ± 19.72 ^h, i, j^
20	80 (1)	0 (−1)	6 (0)	1.43 ± 0.07 ^g, h, i^	239.29 ± 10.51 ^d, e^	327.34 ± 27.20 ^e, f^	239.19 ± 14.59 ^c^	279.16 ± 17.53 ^c, d, e^	16.38 ± 1.48 ^g, h^	359.12 ± 26.16 ^c, d, e^
21	80 (1)	0 (−1)	24 (1)	1.95 ± 0.05 ^c, d, e^	209.33 ± 2.89 ^e, f^	314.25 ± 27.46 ^e, f, g^	246.83 ± 29.19 ^c^	304.24 ± 4.21 ^b, c, d^	8.95 ± 0.80 ^h, i, j^	276.43 ± 17.66 ^f, g, h^
22	80 (1)	20 (0)	3 (−1)	1.83 ± 0.08 ^d, e, f, g^	326.85 ± 8.95 ^b, c^	459.44 ± 42.61 ^b^	340.69 ± 1.70 ^a, b^	263.81 ± 12.79 ^d, e^	36.69 ± 2.39 ^c, d^	530.85 ± 50.88 ^b^
23	80 (1)	20 (0)	6 (0)	1.92 ± 0.10 ^c, d, e, f^	242.53 ± 12.83 ^d^	347.02 ± 26.92 ^d, e^	361.30 ± 36.53 ^a^	274.62 ± 6.79 ^c, d, e^	27.17 ± 2.53 ^e, f^	378.71 ± 3.62 ^c, d^
24	80 (1)	20 (0)	24 (1)	2.67 ± 0.12 ^a, b^	324.98 ± 2.87 ^b, c^	439.91 ± 36.33 ^b, c^	365.79 ± 14.77 ^a^	329.46 ± 28.33 ^a, b, c, d^	74.82 ± 6.57 ^a^	436.89 ± 12.34 ^c^
25	80 (1)	40 (1)	3 (−1)	2.28 ± 0.16 ^b, c, d^	359.87 ± 9.72 ^a^	496.85 ± 44.57 ^a, b^	285.46 ± 21.20 ^b, c^	394.45 ± 36.83 ^a, b, c^	40.87 ± 2.83 ^b, c, d^	863.07 ± 25.67 ^a^
26	80 (1)	40 (1)	6 (0)	2.46 ± 0.10 ^a, b^	341.56 ± 7.11 ^b^	427.55 ± 36.92 ^b, c, d^	290.76 ± 11.12 ^b, c^	427.01 ± 12.12 ^a, b^	74.82 ± 6.57 ^a^	821.22 ± 46.95 ^a^
27	80 (1)	40 (1)	24 (1)	2.85 ± 0.19 ^a^	365.78 ± 1.95 ^a^	519.11 ± 41.19 ^a^	329.59 ± 32.78 ^a, b^	433.75 ± 15.57 ^a^	44.67 ± 4.01 ^b, c^	591.50 ± 42.93 ^b^

X_1_, temperature (°C); X_2_, % EtOH/H_2_O; X_3_, time (h); ^1^ DPPH: 2,2-diphenyl-1-picrylhydrazyl expressed as milligrams of Trolox equivalents per gram of dry extract; ^2^ FRAP: ferric reducing antioxidant power, expressed as milligrams of Trolox equivalents per grams of dry extract; ^3^ TPC: total phenolic content, expressed as milligrams of gallic acid per grams of dry extract; ^4^ TFC: total flavonoid content, expressed as milligrams of quercetin equivalents per grams of dry extract; ^5^ CTC: condensed tannin content, expressed as milligrams of tannic acid equivalents per grams of dry extract; ^6^ HTC: hydrolyzable tannins content, expressed as milligrams of tannic acid equivalents per grams of dry extract. Results are expressed as mean ± standard deviation of mg/g dry weight (DW). In each column, significant differences (*p* < 0.05) between samples are highlighted with different letters (a–m).

**Table 3:** Fit statistics analysis.

Variables	Std. Dev.	Mean	C.V.%	R^2^	Adj. R^2^	Pred. R^2^	Adequate Precision
**^1^ DPPH** **scavenging activity**	48.41	215.54	22.46	0.8262	0.7622	0.6632	12.9832
**^2^** **FRAP**	67.25	317.31	21.20	0.7873	0.7089	0.5342	11.8708
**^3^** **TPC**	50.94	188.65	27.00	0.7503	0.6583	0.5260	9.1674
**^4^** **TFC**	30.05	299.75	10.03	0.7873	0.7090	0.5661	11.9922
**^5^** **CTC**	15.00	26.08	57.51	0.5777	0.4221	−0.1252	7.4330
**^6^** **HTC**	50.94	188.65	27.00	0.7503	0.7906	0.7175	14.2945

^1^ DPPH: 2,2-diphenyl-1-picrylhydrazyl expressed as milligrams of Trolox equivalents per gram of dry extract; ^2^ FRAP: ferric reducing antioxidant power, expressed as milligrams of Trolox equivalents per grams of dry extract; ^3^ TPC: total phenolic content, expressed as milligrams of gallic acid per grams of dry extract; ^4^ TFC: total flavonoid content, expressed as milligrams of quercetin equivalents per grams of dry extract; ^5^ CTC: condensed tannin content, expressed as milligrams of tannic acid equivalents per grams of dry extract; ^6^ HTC: hydrolyzable tannins content, expressed as milligrams of tannic acid equivalents per grams of dry extract; C.V.%: coefficient of variation.

**Table 4:** Linear regression equations and statistical analysis.

Response	Independent Variables								
**^1^** **DPPH**	Y_DPPH_ =	+215.49	+83.26 X_1_	+70.72 X_2_	−0.22 X_3_	−10.00 X_1_ X_2_	+9.77 X_1_ X_3_	+5.37 X_2_ X_3_	+17.28 X_1_ X_2_ X_3_
***p*-values**			<0.0001 *	<0.0001 *	0.9839	0.4981	0.4590	0.6822	0.2885

**^2^** **FRAP**	Y_FRAP_ =	+318.45	+99.05 X_1_	+86.16 X_2_	+4.82 X_3_	−20.49 X_1_ X_2_	−0.58 X X_3_	+28.64 X_2_ X_3_	+27.26 X_1_ X_2_ X_3_
***p*-values**			<0.0001 *	<0.0001 *	0.7458	0.3210	0.9745	0.1271	0.2301

**^3^** **TPC**	Y_TPC_ =	+190.90	+86.75 X_1_	+27.04 X_2_	+9.47 X_3_	+2.81 X_1_ X_2_	+2.79 X_1_ X_3_	+3.22 X_2_ X_3_	+3.83 X_1_ X_2_ X_3_
***p*-values**			<0.0001 *	0.0425 *	0.4041	0.8558	0.8396	0.8152	0.8206

**^4^** **TFC**	Y_TFC_ =	+305.96	+31.41 X_1_	+34.52 X_2_	+26.07 X_3_	+21.15 X_1_ X_2_	−7.89 X_1_ X_3_	−5.19 X_2_ X_3_	+6.09 X_1_ X_2_ X_3_
***p*-values**			0.0004 *	0.0002 *	0.0008 *	0.0295 *	0.3374	0.5253	0.5427

**^5^** **HTC**	Y_HTC_ =	+310.52	+152.80 X_1_	+101.96 X_2_	−48.99 X_3_	+105.06 X_1_ X_2_	−1.61 X_1_ X_3_	−47.88 X_2_ X_3_	−8.10 X_1_ X_2_ X_3_
***p*-values**			<0.0001 *	0.0002 *	0.0234 *	0.0011 *	0.9478	0.0629	0.7886

^1^ DPPH scavenging activity: 2,2-diphenyl-1-picrylhydrazyl; ^2^ FRAP: ferric reducing antioxidant power ^3^ TPC: total phenolic content; ^4^ TFC: total flavonoid content; ^5^ HTC: hydrolyzable tannins content. X_1_, X_2_, and X_3_ represent the three variables (temperature, % EtOH/H_2_O, and time, respectively). * Variables with a *p*-value < 0.05 are significant.

**Table 5:** Predicted results from response optimization.

Methods	Response Prediction	95% PI ^a^	SE Fit ^b^
DPPH ^1^ scavenging activity	378.7 mg TE/g	(257.2; 500.2)	32.1
FRAP ^2^	519.1 mg TE/g	(350.3; 688.0)	44.5
TPC ^3^	319.0 mg GAE/g	(191.2; 446.9)	33.7
TFC ^4^	404.4 mg QE/g	(329.0; 479.9)	19.9
HTC ^5^	606.6 mg TAE/g	(377.7; 835.6)	60.4

^1^ DPPH: 2,2-diphenyl-1-picrylhydrazyl, expressed as milligrams of Trolox equivalents per gram of dry extract; ^2^ FRAP: ferric reducing antioxidant power, expressed as milligrams of Trolox equivalents per grams of dry extract; ^3^ TPC: total phenolic content, expressed as milligrams of gallic acid per grams of dry extract; ^4^ TFC: total flavonoid content, expressed as milligrams of quercetin equivalents per grams of dry extract, ^5^ HTC: hydrolyzable tannins content, expressed as milligrams of tannic acid equivalents per grams of dry extract. ^a^ Prediction interval. ^b^ Standard error of the fit.

**Table 6:** Pearson correlation values.

	^3^ TPC (mg GAE/g)	^4^ TFC (mg QE/g)	^5^ HTC (mg TAE/g)	FRAP (mg TE/g)
TFC (mg QE/g)	0.484			
HTC (mg TAE/g)	0.661	0.621		
^2^ FRAP (mg TE/g)	0.733	0.576	0.601	
^1^ DPPH scavenging activity (mg TE/g)	0.737	0.597	0.726	0.931

^1^ DPPH: 2,2-diphenyl-1-picrylhydrazyl, expressed as milligrams of Trolox equivalents per gram of dry extract; ^2^ FRAP: ferric reducing antioxidant power, expressed as milligrams of Trolox equivalents per grams of dry extract; ^3^ TPC: total phenolic content, expressed as milligrams of gallic acid per grams of dry extract; ^4^ TFC: total flavonoid content, expressed as milligrams of quercetin equivalents per grams of dry extract; ^5^ HTC: hydrolyzable tannins content, expressed as milligrams of tannic acid equivalents per grams of dry extract.

**Table 7:** Comparison between FFD and ANN models’ predictive capability.

			^1^ DPPH Scavenging Activity (mg TE/g)			^2^ FRAP (mg TE/g)			^3^ TPC (mg GAE/g)		
Temperature	% EtOH	Time	Exp.	FFD	ANN	Exp.	FFD	ANN	Exp.	FFD	ANN
20	0	3	72.69	49.58	80.03	134.32	108.73	124.03	104.45	72.63	107.60
20	0	6	92.66	50.13	90.91	145.65	109.88	154.68	105.18	74.71	100.55
20	0	24	41.88	53.43	50.56	135.10	116.78	173.86	114.46	87.21	70.00
20	20	3	120.78	142.21	93.88	215.87	214.00	144.68	103.87	97.47	130.31
20	20	6	116.87	139.36	119.72	196.71	215.54	207.41	107.29	99.38	123.91
20	20	24	112.53	122.25	117.40	199.78	224.81	217.71	121.82	110.84	137.75
20	40	3	220.89	234.83	236.11	252.48	319.27	274.15	150.76	122.32	158.90
20	40	6	308.96	228.58	319.80	474.09	321.21	429.46	167.19	124.05	158.94
20	40	24	165.69	191.06	205.53	303.11	332.83	313.00	169.16	134.47	180.55
50	0	3	104.49	150.35	121.93	190.43	256.11	186.74	98.82	157.61	110.35
50	0	6	195.99	148.75	165.07	241.46	249.31	249.51	122.95	159.39	111.00
50	0	24	165.69	139.18	116.96	214.05	208.48	202.20	131.17	170.11	131.99
50	20	3	112.44	215.70	141.92	316.91	313.63	225.96	139.18	181.43	120.49
50	20	6	165.69	215.64	168.19	250.94	315.01	255.13	142.38	184.14	131.82
50	20	24	203.64	215.27	218.12	285.25	323.27	273.73	143.95	200.37	146.86
50	40	3	322.41	281.06	311.50	362.79	371.15	364.13	148.16	205.25	155.19
50	40	6	238.77	282.53	326.93	287.31	380.71	393.38	152.97	208.88	165.17
50	40	24	347.71	291.37	341.79	518.48	438.07	485.69	175.98	230.63	233.86
80	0	3	299.60	251.12	314.86	511.04	403.49	494.92	234.04	242.59	241.04
80	0	6	239.29	247.38	231.34	327.34	388.73	344.60	239.19	244.08	233.94
80	0	24	209.33	224.92	212.08	314.25	300.18	315.31	246.83	253.01	249.10
80	20	3	326.85	289.20	258.35	459.44	413.26	381.11	340.69	265.39	338.50
80	20	6	242.53	291.93	249.25	347.02	414.48	341.47	361.30	268.89	364.56
80	20	24	324.98	308.30	315.08	439.91	421.74	445.88	365.79	289.91	358.56
80	40	3	359.87	327.28	368.56	496.85	423.04	489.75	285.46	288.18	282.63
80	40	6	341.56	336.48	337.29	427.55	440.22	435.94	290.76	293.70	290.92
80	40	24	365.78	391.67	359.10	519.11	543.31	526.07	329.59	338.99	340.89
**Model-predicted capability**											
				**^1^ DPPH scavenging activity**			**^2^ FRAP**			**^3^ TPC**	
				**FFD**	**ANN**		**FFD**	**ANN**		**FFD**	**ANN**
**^4^** **MAE**				33.92	18.43		43.22	24.12		34.63	11.30
**^5^** **RMSE**				40.61	27.72		56.42	37.34		42.73	17.17
**^6^** **R^2^**				0.83	0.92		0.79	0.96		0.75	0.91

^1^ DPPH: 2,2-diphenyl-1-picrylhydrazyl, expressed as milligrams of Trolox equivalents per gram of dry extract; ^2^ FRAP: ferric reducing antioxidant power, expressed as milligrams of Trolox equivalents per grams of dry extract; ^3^ TPC: total phenolic content, expressed as milligrams of gallic acid per grams of dry extract; ^4^ MAE mean absolute error; ^5^ RMSE: root mean square error, ^6^ R^2^: coefficient of determination; Exp.: experimental; FFD: full factorial design; ANN: artificial neural network.

**Table 8:** FFD or ANN models’ extraction condition optimization.

	Temperature °C	Time h	% EtOH/H_2_O	^1^ DPPH Scavenging Activity mg TE/g	^2^ FRAP mg TE/g	^3^ TPC mg GAE/g	Composite Desirability
**FFD**	80.00	24.00	40.00	391.67	543.31	326.80	0.95
**ANN**	80.00	23.47	39.32	380.22	526.22	357.24	0.99

^1^ DPPH: 2,2-diphenyl-1-picrylhydrazyl, expressed as milligrams of Trolox equivalents per gram of dry extract; ^2^ FRAP: ferric reducing antioxidant power, expressed as milligrams of Trolox equivalents per grams of dry extract; ^3^ TPC: total phenolic content, expressed as milligrams of gallic acid per grams of dry extract; FFD: full factorial design; ANN: artificial neural network.

## Data Availability

Data are available upon request.

## Supplementary Materials

The following supporting information can be downloaded at: https://www.mdpi.com/article/10.3390/antiox13091115/s1, Table S1. Analysis of variance of DPPH, FRAP, TPC, TFC, CTC, and HTC methods; Table S2. Pearson correlation between yield and the evaluated responses; Table S3: Weights and biases of the developed ANN; Figure S1 Performance of ANN model with different numbers of hidden layers (A) 8, (B) 9, (C) 10, (D) 11, and (E) 12.
