# Supplementary material for: Extraction Optimization of Quercus cerris L. Wood Chips: A Comparative Study between Full Factorial Design (FFD) and Artificial Neural Network (ANN)
Source: Antioxidants (Basel). 2024 Sep 14;13(9):1115. doi: 10.3390/antiox13091115 (PMC11428725; doi:10.3390/antiox13091115)
Supplement: Supplementary file 1 [file antioxidants-13-01115-s001.zip › antioxidants-3136278-supplementary.pdf]

Article: Supplementary material

Extraction optimization of *Quercus cerris* L. wood chips: a comparative study between Full Factorial Design (FFD) and Artificial Neural Network (ANN)

Maria Ponticelli [1,2,†], Vittorio Carlucci [1,†], Marisabel Mecca [3], Luigi Todaro [4], Luigi Milella [1,*] and Daniela Russo [1,5]

[1] Department of Science, University of Basilicata, Via dell'Ateneo Lucano 10, 85100 Potenza, Italy; maria.ponticelli@unibas.it (M.P.); vittorio.carlucci@unibas.it (V.C.); daniela.russo@unibas.it (D.R.)
[2] Department of Biochemical Pharmacology & Drug Design, Institute of Molecular Biology "Roumen Tsanev", Bulgarian Academy of Sciences (BAS), Acad. G. Bonchev Str., bl. 21, 1113 Sofia, Bulgaria
[3] Laboratory of Preclinical and Translational Research, Centro di Riferimento Oncologico della Basilicata (IRCCS-CROB), 85028 Rionero in Vulture, Italy; marisabelmecca@libero.it (M.M)
[4] School of Agriculture, Forest, Food and Environmental Sciences, University of Basilicata, Via dell'Ateneo Lucano 10, 85100 Potenza, Italy; luigi.todaro@unibas.it (L.T.)
[5] BioActiPlant s.r.l., Via dell'Ateneo Lucano 10, 85100 Potenza, Italy
* Correspondence: luigi.milella@unibas.it (L.M.); Tel./Fax: +39-0971205525
† These authors contributed equally to this work.

Table S1. Analysis of variance of DPPH, FRAP, TPC, TFC, CTC and HTC methods
Table S2 Pearson Correlation between yield and the evaluated responses
Table S3: Weights and biases of the developed ANN

Figure S1 Performance of ANN model with different numbers of hidden layers A) 8, B) 9, C) 10, D) 11, and E) 12

Table S1. Analysis of variance of DPPH, FRAP, TPC, TFC, CTC and HTC methods

Source	DF	Adj SS	Adj MS	F-Value	P-Value
**DPPH scavenging activity**					
**Model**	7	211646	30235	12.90	0.000 *
**Linear**	3	200264	66755	28.49	0.000 *
**Temperature (°C)**	1	116327	116327	49.65	0.000 *
**Solvent (%EtOH)**	1	83936	83936	35.82	0.000 *
**Time(h)**	1	1	1	0.00	0,984
**2-Way Interactions**	3	2862	954	0.41	0.750
**Temperature (°C)*Solvent (%EtOH)**	1	1118	1118	0.48	0.498
**Temperature (°C)*Time(h)**	1	1339	1339	0.57	0.459
**Solvent (%EtOH)*Time(h)**	1	405	405	0.17	0.682
**3-Way Interactions**	1	2793	2793	1.19	0.289
**Temperature (°C)*Solvent (%EtOH)*Time(h)**	1	2793	2793	1.19	0.289
**Error**	19	44518	2343		
**Total**	26	256164			
**FRAP**					
**Model**	7	318097	45442	10.05	0.000 *
**Linear**	3	289690	96563	21.35	0.000 *
**Temperature (°C)**	1	164633	164633	36.40	0.000 *
**Solvent (%EtOH)**	1	124568	124568	27.54	0.000 *
**Time(h)**	1	489	489	0.11	0.746
**2-Way Interactions**	3	16215	5405	1.19	0.338
**Temperature (°C)*Solvent (%EtOH)**	1	4696	4696	1.04	0.321
**Temperature (°C)*Time(h)**	1	5	5	0.00	0.974
**Solvent (%EtOH)*Time(h)**	1	11514	11514	2.55	0.127
**3-Way Interactions**	1	6954	6954	1.54	0.230
**Temperature (°C)*Solvent (%EtOH)*Time(h)**	1	6954	6954	1.54	0.230
**Error**	19	85940	4523		
**Total**	26	404037			
**TPC**					
**Model**	7	148131	21162	8.16	0.000 *
**Linear**	3	140430	46810	18.04	0.000 *
**Temperature (°C)**	1	126270	126270	48.67	0.000 *
**Solvent (%EtOH)**	1	12271	12271	4.73	0.042 *
**Time(h)**	1	1889	1889	0.73	0.404
**2-Way Interactions**	3	343	114	0.04	0.987
**Temperature (°C)*Solvent (%EtOH)**	1	88	88	0.03	0.856
**Temperature (°C)*Time(h)**	1	109	109	0.04	0.840
**Solvent (%EtOH)*Time(h)**	1	146	146	0.06	0.815
**3-Way Interactions**	1	137	137	0.05	0.821
**Temperature (°C)*Solvent (%EtOH)*Time(h)**	1	137	137	0.05	0.821

**Error**	19	49293	2594		
**Total**	26	197424			
			**TFC**		
**Model**	7	63538.1	9076.9	10.05	0.000 *
**Linear**	3	50871.1	16957.0	18.77	0.000 *
**Temperature (°C)**	1	16551.4	16551.4	18.32	0.000 *
**Solvent (%EtOH)**	1	20000.7	20000.7	22.14	0.000 *
**Time(h)**	1	14319.0	14319.0	15.85	0.001 *
**2-Way Interactions**	3	6258.5	2086.2	2.31	0.109
**Temperature (°C)*Solvent (%EtOH)**	1	5005.2	5005.2	5.54	0.029 *
**Temperature (°C)*Time(h)**	1	875.1	875.1	0.97	0.337
**Solvent (%EtOH)*Time(h)**	1	378.2	378.2	0.42	0.525
**3-Way Interactions**	1	347.0	347.0	0.38	0.543
**Temperature (°C)*Solvent (%EtOH)*Time(h)**	1	347.0	347.0	0.38	0.543
**Error**	19	17162.1	903.3		
**Total**	26	80700.1			
			**HTC**		
**Model**	7	874460	124923	15.03	0.000 *
**Linear**	3	616807	205602	24.73	0.000 *
**Temperature (°C)**	1	391807	391807	47.13	0.000 *
**Solvent (%EtOH)**	1	174462	174462	20.99	0.000 *
**Time (h)**	1	50538	50538	6.08	0.023 *
**2-Way Interactions**	3	155697	51899	6.24	0.004 *
**Temperature (°C)*Solvent (%EtOH)**	1	123467	123467	14.85	0.001 *
**Temperature (°C)*Time(h)**	1	37	37	0.00	0.948
**Solvent (%EtOH)*Time(h)**	1	32194	32194	3.87	0.064
**3-Way Interactions**	1	615	615	0.07	0.789
**Temperature (°C)*Solvent (%EtOH)*Time(h)**	1	615	615	0.07	0.789
**Error**	19	157959	8314		
**Total**	26	1032418			
			**CTC**		
**Model**	7	5848.2	835.46	3.71	0.011 *
**Linear**	3	5641.1	1880.36	8.36	0.001 *
**Temperature (°C)**	1	2642.0	2641.96	11.74	0.003 *
**Solvent (%EtOH)**	1	2999.1	2999.12	13.33	0.002 *
**Time(h)**	1	0.0	0.00	0.00	0.999
**2-Way Interactions**	3	263.5	87.83	0.39	0.761
**Temperature (°C)*Solvent (%EtOH)**	1	69.4	69.37	0.31	0.585
**Temperature (°C)*Time(h)**	1	8.9	8.91	0.04	0.844
**Solvent (%EtOH)*Time(h)**	1	185.2	185.22	0.82	0.376
**3-Way Interactions**	1	5.3	5.30	0.02	0.880
**Temperature (°C)*Solvent (%EtOH)*Time(h)**	1	5.3	5.30	0.02	0.880

**Error**		19	4275.0	225.00
**Total**		26	10123.2	

DF, degrees of freedom; SS, sum of squares; MS, mean squares; R^2, coefficient of determination; DPPH: 2,2-diphenyl-1-picrylhydrazyl expressed as milligrams of Trolox equivalents per gram of dry extract; FRAP: ferric reducing antioxidant power expressed as milligrams of Trolox equivalents per grams of dry extract. TPC: total phenolic content expressed as milligrams of gallic acid per gram of dry extract; TFC: total flavonoid content expressed as milligrams of quercetin equivalents per grams of dry extract; CTC: condensed tannin content expressed as milligrams of tannic acid equivalents per grams of dry extract; HTC: hydrolysable tannins content expressed as milligrams of tannic acid equivalents per grams of dry extract; * p values lower than 0.05 are statistically significant.

Table S2. Pearson Correlation between yield and the evaluated responses

	Temperature (°C)	TPC (mgGAE/g)	TFC (mgQE/g)	HTC (mgTAE/g)	FRAP (mgTE/g)	DPPH (mgTE/g)
**Yield (%)**	0.774	0.811	0.787	0.669	0.779	0.791
**95%CI**	(0.558;0.892)	(0.623;0.910)	(0.581;0.898)	(0.387;0.836)	(0.567;0.894)	(0.587;0.900)

DPPH: 2,2-diphenyl-1-picrylhydrazyl expressed as milligrams of Trolox equivalents per gram of dry extract; FRAP: ferric reducing antioxidant power expressed as milligrams of Trolox equiva-lents per grams of dry extract. TPC: total phenolic content expressed as milligrams of gallic acid per gram of dry extract; TFC: total flavonoid content expressed as milligrams of quercetin equivalents per grams of dry extract; CTC: condensed tannin content expressed as milligrams of tannic acid equivalents per grams of dry extract; HTC: hydrolysable tannins content expressed as milligrams of tannic acid equivalents per grams of dry extract

Table S2: Weights and biases of the developed ANN

**Layer 1**
b1 = [-3.3658381272721920041; -1.3991178379491064909; 0.73659995576991865729; -0.53182049141105114387; 0.52472355555572924857; -2.9733963321470882057; -0.29085727691318830068; -2.4964822118479084168; -2.8047356204451689976;-3.2135318183290730865];
IW1_1 = [1.3694162100906195345 -4.3091618142965266713 1.1935791040671703822; 2.8121322381007556324 -1.5218647632018840188 0.041052536626025579181; 1.2410943326698309352 4.4725625112068927436 -2.1986119377570134681; 1.7992859703924799497 -0.54371115367242317529 -4.456133682056255374; 1.6843631748729901432 -0.27292574815673481536 3.9010079034444609114; -3.5315655357644906154 0.011234577183720562132 -0.025679126289264435357; -0.95195687849418597626 -2.4057537243378814829 -1.1475221339558825395; -2.210554458180312043 1.7111592469016598805 -0.58368623918766127101; -2.9614786203794487562 0.11948928895135463302 0.17259416977169131369; 0.41276498321934224078 -2.3323459078827615265 1.9361919122015875949];
**Layer 2**
b2 = [-0.51842244257765668358;-0.66108449055888895973;-0.54882049544378741501];
LW2_1 = [0.94671609759769825843 0.057486181383335541173 0.34417166533069570855 -0.6916455621694962419 -0.74373535304976634563 1.1331329958826239501 -0.8161099264387802199 -0.3444863845680172787 -1.4062761166421580139 -0.7058946673556334428; 1.1952698840557116622 0.13209506421923833397 0.53609279801075515604 -1.1339154431549203217 -1.0731688872653484257 1.0415563925734900863 -0.76866176993251578153 -0.67120669180756709071 -0.9821873938496097356 -0.92983195083105829593; 0.048017442413148953007 0.73243565977217839347 0.38052041677848280621 0.029593823126837982829 0.18298537281625720796 1.2230096454784413762 -0.38715958756250995476 -0.38143242427172541209 -1.1342958393706217279 -0.18005823578382687544];

b1: Bias values for hidden layer neurons;
IW1_1: Weights of synaptic joints between input and hidden layer neurons;
b2: Bias values for hidden layer neurons.
LW2_1: Weights of synaptic joints between hidden and output layer neurons;

A

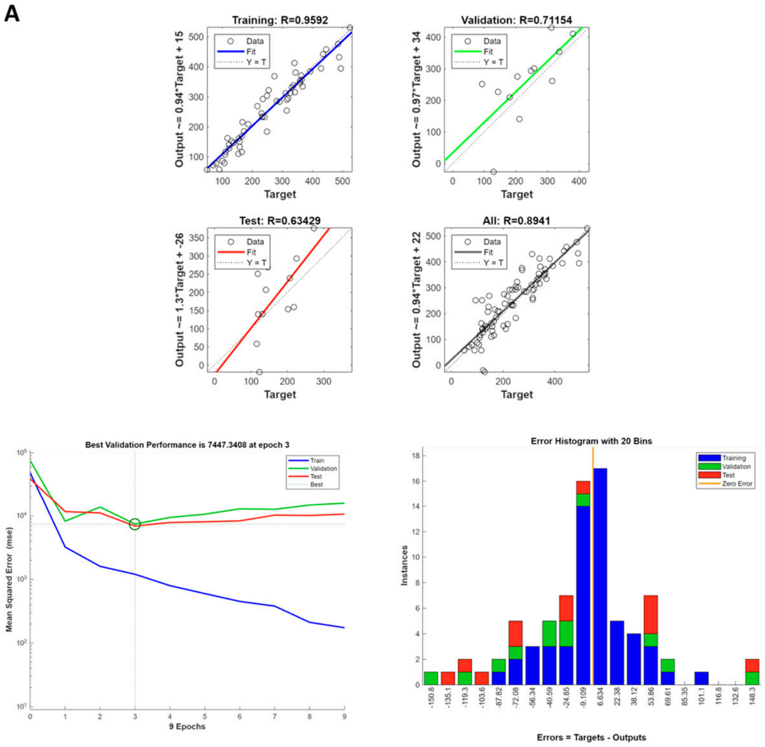

Train a neural network to map predictors to continuous responses.

Data

Predictors: X - [27x3 double]

Responses: Y - [27x3 double]

X: double array of 27 observations with 3 features.

Y: double array of 27 observations with 3 features.

Algorithm

Data division: Random

Training algorithm: Levenberg-Marquardt

Performance: Mean squared error

Training Results

Layer size: 8

	Observations	MSE	R
**Training**	19	1.2067e+03	0.9592
**Validation**	4	7.4473e+03	0.7115
**Test**	4	6.9241e+03	0.6343

B

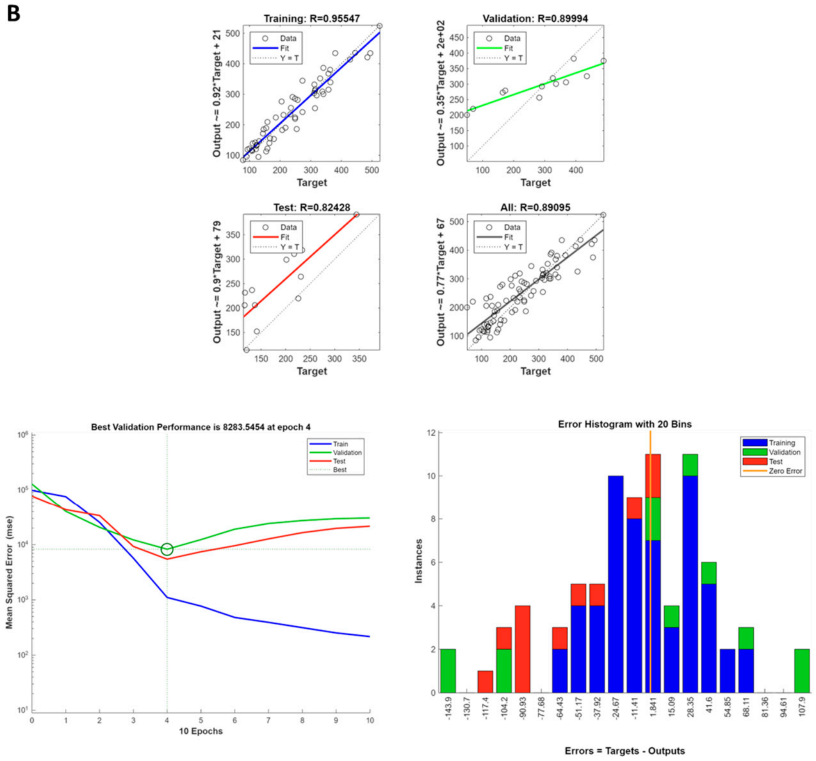

Train a neural network to map predictors to continuous responses.

Data

Predictors: X - [27x3 double]

Responses: Y - [27x3 double]

X: double array of 27 observations with 3 features.

Y: double array of 27 observations with 3 features.

Algorithm

Data division: Random

Training algorithm: Levenberg-Marquardt

Performance: Mean squared error

Training Results

Layer size: 9

	Observations	MSE	R
**Training**	19	1.1070e+03	0.9555
**Validation**	4	8.2835e+03	0.8999
**Test**	4	5.4680e+03	0.8243

C

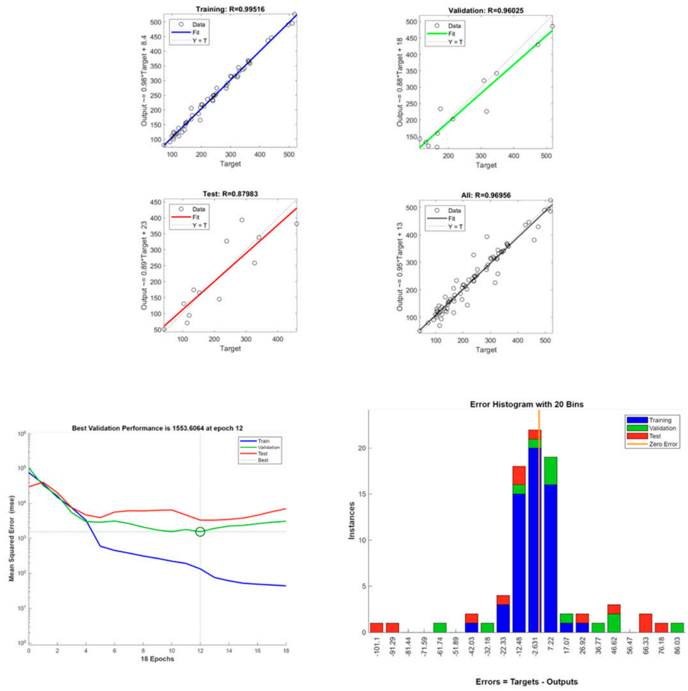

Model Summary

Train a neural network to map predictors to continuous responses.

Data

Predictors: x - [3x27 double]

Responses: y - [3x27 double]

x: double array of 27 observations with 3 features.

y: double array of 27 observations with 3 features.

Algorithm

Data division: Random

Training algorithm: Levenberg-Marquardt

Performance: Mean squared error

Training Results

Layer size: 10

	Observations	MSE	R
**Training**	19	134.4447	0.9952
**Validation**	4	1.5536e+03	0.9603
**Test**	4	3.3374e+03	0.8798

D

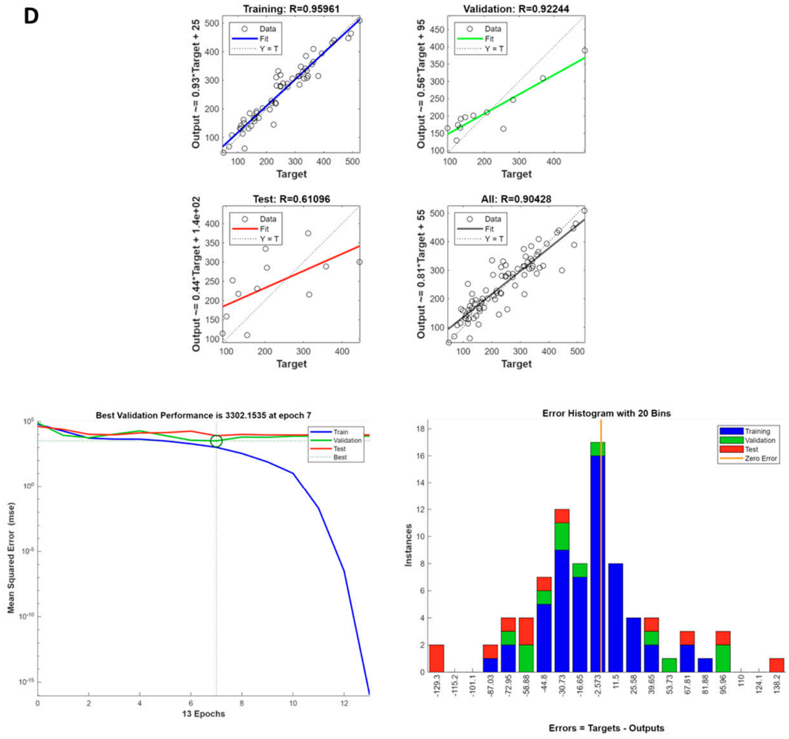

Train a neural network to map predictors to continuous responses.

Data

Predictors: X - [27x3 double]

Responses: Y - [27x3 double]

X: double array of 27 observations with 3 features.

Y: double array of 27 observations with 3 features.

Algorithm

Data division: Random

Training algorithm: Levenberg-Marquardt

Performance: Mean squared error

Training Results

Layer size: 11

	Observations	MSE	R
**Training**	19	1.0518e+03	0.9596
**Validation**	4	3.3022e+03	0.9224
**Test**	4	8.1649e+03	0.6110

E

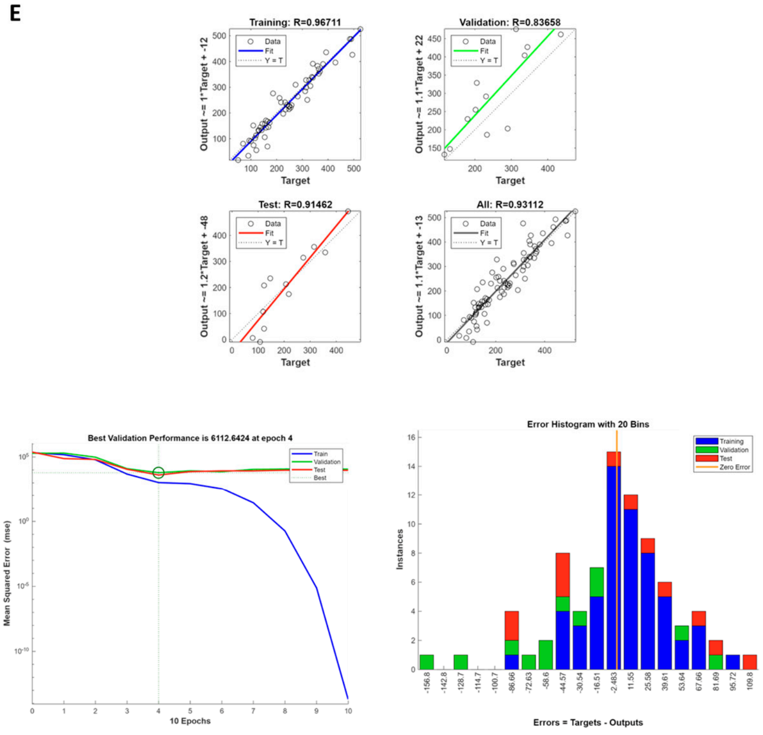

Train a neural network to map predictors to continuous responses.

Data

Predictors: X - [27x3 double]

Responses: Y - [27x3 double]

X: double array of 27 observations with 3 features.

Y: double array of 27 observations with 3 features.

Algorithm

Data division: Random

Training algorithm: Levenberg-Marquardt

Performance: Mean squared error

Training Results

Layer size: 12

	Observations	MSE	R
**Training**	19	1.0562e+03	0.9671
**Validation**	4	6.1126e+03	0.8366
**Test**	4	4.0670e+03	0.9146

Figure S1 Performance of ANN model with different numbers of hidden layers A) 8, B) 9, C) 10, D) 11, and E) 12
